# Description and Pathogenicity of *Colletotrichum kapreanum* sp. nov, a Cherelle Wilt Pathogen Belonging to the Gigasporum Species Complex

**DOI:** 10.3390/jof10030204

**Published:** 2024-03-08

**Authors:** Yoshiki Takata, Celynne Ocampo-Padilla, Mike Andre C. Malonzo, Dan Charlie Joy Camara Pangilinan, Shunsuke Nozawa, Kyoko Watanabe

**Affiliations:** 1 Graduate School of Agriculture, Tamagawa University, Tamagawa-Gakuen 6-1-1, Machida 194-8610, Japancelynnepadilla@clsu.edu.ph (C.O.-P.); dcjcpangilinan@clsu.edu.ph (D.C.J.C.P.); 2 Department of Crop Protection, Central Luzon State University, Science City of Muñoz 3120, Philippines; 3 BaCaDM Project, College of Agriculture, Tamagawa University, Tamagawa-Gakuen 6-1-1, Machida 194-8610, Japan; mikeandremalonzo@gmail.com; 4 College of Agriculture, Tamagawa University, Tamagawa-Gakuen 6-1-1, Machida 194-8610, Japan; nozawa@agr.tamagawa.ac.jp

**Keywords:** cherelle wilt, cacao pod rot, anthracnose, *Theobroma cacao* L., *Colletotrichum kapreanum*, gigasporum species complex, new species

## Abstract

Similar to cacao pod rot, cherelle wilt decreases production from cacao fields. Among all known fungal pathogens of the cacao, *Colletotrichum* spp. are common infectious agents that affect the cherelles and pods of cacao; thus, cacao diseases are often classified by stage. Therefore, knowing whether these pathogens are common in both fruit stages is necessary for implementing disease control measures. Symptoms of cherelle wilt were found in cacao plants in Pangasinan, Philippines, in 2022. The fungal strain obtained from the lesion was found to be pathogenic towards cherelles, but not towards pods. The strain was classified as an unknown species belonging to the gigasporum species complex, based on the morphological and molecular phylogenetic analyses of ITS, *GAPDH*, *CHS1*, *ACT*, and *TUB2*. We propose *Colletotrichum kapreanum* sp. nov. as a causal agent of cacao cherelle wilt, but not pod rot.

## 1. Introduction

Cacao (*Theobroma cacao* L.), the source of cacao beans used to make chocolate, is an important cash crop in tropical countries [1,2]. In the Philippines, the majority of the cacao farms are smallholdings owned and managed by farmers. According to Philippine Statistics Authority (PSA) data, the Philippines’ cacao production has been slowly but steadily increasing, by an average of 2743 hectares per year, from 2013 to 2020. Though production is increasing, the country cannot still meet the increasing market demand of cacao locally and globally. The low production of cacao in the Philippines is mainly due to the high mortality rate of planting materials and the lack of knowledge and training specifically on good agricultural practices (GAP) for cacao farmers, resulting in improper farm management, which can result in low yield and susceptibility of cacao plants to pests and diseases [3].

Cacao pods are classified by size, into either ‘cherelles’ and ‘pods.’ Cherelles are defined as pods with a length <10 cm, while pods are >10 cm. Cacao diseases are classified based on the pod stage or morphology. Cacao pod rot is caused by several pathogens; thus, it is difficult to identify based solely on its symptoms. However, some pathogens that cause diseases in pods have their own disease names; for example, black pod rot and brown pod rot are caused by *Phytophthora* spp.; frosty pod disease is caused by *Moniliophthora roreri* (Cif.) H.C. Evans, Stalpers, Samson & Benny; and anthracnose is caused by *Colletotrichum* spp., all of which are diseases that have generated research interest. However, 80% of cherelle wilt cases are thought to be caused by physiological disorders [2]; thus, little research has been conducted on the relevant infections. Therefore, cherelle wilt is a disease that farmers are unfamiliar with as a control target.

Anthracnose disease in cacao, caused by *Colletotrichum* spp., manifests as lesions on the leaves and pods [4,5]. To date, all recorded causative species belong to the *Colletotrichum gloeosporioides* species complex (CGLSC), which comprises *C. gloeosporioides*, *C. siamense*, *C. theobromicola*, *C. tropicale*, and *C. aeschynomenes* [4,6,7,8,9]. Among these, *C. tropicale* is pathogenic to cacao pods at different maturation stages [5], and *C. theobromicola* Delacr. has been isolated from both cherelle wilt and cacao pod rot [1]. There is no record of other species as common pathogens in cherelles and pods. Thus, there may be a low potential for pathogens in cherelles and pods to infect each other and cause anthracnose outbreaks in cacao-producing farms. Additionally, many diseases from *Colletotrichum* species are reported as pathogens causing disease on fruits and leaves, such as diseases associated with *C. gloeosporioides* (syn. *Glomerella cingulate*), which causes leaf spots on apples [10]; *C. truncatum*, which causes leaf spots on leaves of *Citrus reticulata*; [11] and *C. acutatum*, which causes almond anthracnose [12]. However, González et al. [10] found that some of the lineages of *C. acutatum* and *C. gloeosporioides* show host organ-specific pathogenicity. Some studies show that the latent phase of pathogenic *Colletotrichum* spp. in fruits, such as apples, strawberries, and citrus, is present in the leaves and twigs of the plant. In cacao, Rojas et al. [9] reported that fungi belonging to the CGLSC and gigasporum species complex (CGISC) have the potential to act not only as endophytes, but also as pathogens. Therefore, these pathogens are prospective targets for control of the disease.

This study aimed to identify the pathogen causing anthracnose in cacao cherelles and to determine its pathogenicity to cherelles, pods, and leaves, as well as to elucidate its life cycle for disease control strategies. We identified an isolate, PH22T135, obtained from a cherelle wilt at the species level based on morphological and molecular phylogenetic analysis, and tested its pathogenicity to cherelles, pods, and leaves of cacao. The strain was thus identified as a new species belonging to the CGISC and pathogenic only to cherelles.

## 2. Materials and Methods

### 2.1. Fungal Isolation

**Figure 1:**
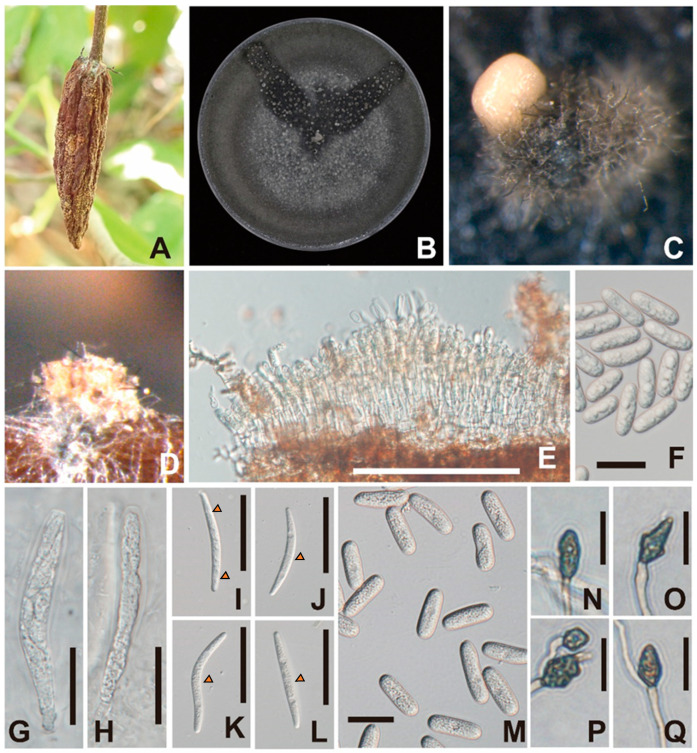
Cacao cherelle with symptoms of cherelle wilt (**A**). Colony of pathogenic PH22T135 on OA plate (**B**). Mucilaginous conidial mass on an acervulus developed on an OA plate (**C**). Conidial mass and acervulus developed on pine needles (**D**). Longitudinal section of an acervus; conidia were produced from conidiogenesis cells lined inside the acervulus on a pine needle (**E**). Conidia on a pine needle (**F**). Ascus containing unmatured ascospores on an OA plate (**G**,**H**). Ascospores; arrowheads point to septa (**I**–**L**). Conidia on an OA plate (**M**). Appressoria produced inside of the SNA plate (**N**–**Q**). Scale bars: (**E**,**G**–**L**) = 50 μm, (**F**,**M**) = 20 μm, (**N**–**Q**) = 10 μm.

A cacao fruit sample showing “cherelle wilt” was collected in June 2022 from Pangasinan, Philippines. The fruit was blighted and dried with salmon-pink conidiomata (Figure 1A). Approximately 5 × 5 mm segments of symptomatic pod tissues were soaked into 0.6% (*v*/*v*) sodium hypochlorite for 30 s for surface sterilization, blot-dried using sterile tissue paper, and placed on water agar (agar 15 g/L) plates. Hyphae emerging from the tissues were transferred onto potato dextrose agar (PDA; 39 g/L; Eiken Kagaku, Tokyo, Japan) plates and maintained at room temperature (25 °C) to induce sporulation. A monoculture was obtained by isolating a single conidium from a conidial mass of the fungal colony. The isolate was maintained on a PDA plate. Our isolate was preserved in a tube with 10% sterile glycerol at −80 °C, as the ex-type living culture and type specimen were deposited at Central Luzon State University.

### 2.2. Morphological Observation

After a ten-day inoculation period, the characteristics of colonies on oatmeal agar (OA; oatmeal 20 g/L, agar 15 g/L) plate and synthetic nutrient-poor agar (SNA) [13] were observed at 25 °C in natural light conditions. Conidial masses produced on OA plates and pine needle placed on SNA plates were observed under an MDG-17 stereo microscope (Leica Microsystems GmbH, Wetzlar, Germany). Morphological characteristics of asci, ascospores, and conidia on OA plates or pine needles placed on SNA plates as well as the appressoria that formed on the bottom surface of the SNA plates were examined under a light microscope (BX51, Olympus, Tokyo, Japan). Each structure (*n* = 30) was measured using ImageJ software 1.53o (https://imagej.nih.gov/ij/index.html, accessed on 20 January 2022).

### 2.3. Genomic DNA Extraction, PCR, and Sequencing

DNA was extracted for polymerase chain reaction (PCR) using the cetyltrimethylammonium bromide method described by Doyle and Doyle [14]. Complete sequences of the internal transcribed spacer 1, 2 including 5.8S rRNA, and partial sequences of 18S rRNA and 28S rRNA regions (ITS), *glyceraldehyde-3-phosphate dehydrogenase* (*GAPDH*), *chitin synthase 1* (*CHS1*), *actin* (*ACT*), and *β-tubulin 2* (*TUB2*) genes were amplified using the primer pairs ITS1F [15]/ITS4 [16], GDF/GDR [17], CoCHS586F/CoCHS2089R [18], CoACTF/CoACTR [18], and CoTUB2F/CoTUB2R [18], respectively. The PCR conditions for amplifying these gene regions were in accordance with the report of Liu et al. [19]. PCR products were purified using ExoSAP-IT reagent (GE Healthcare, Tokyo, Japan) after detecting bands using electrophoresis. Cycle sequencing reactions were conducted using BigDye Terminator v3.1 (Applied Biosystems, Tokyo, Japan). Purified sequencing products obtained by ethanol precipitation were analyzed at FASMAC DNA Sequencing Services (Kanagawa, Japan).

### 2.4. Molecular Phylogenetic Analyses

To identify PH22T135, 18 sequences of strains belonging to 10 species under the CGISC were used to construct molecular phylogenetic trees (Table 1). Sequences from *Colletotrichum acutatum* (CBS112996) and *C. gloeosporioides* (ICMP17821) were used as outgroups. The sequences of ITS, *GAPDH*, *CHS1*, *ACT*, and *TUB2* were aligned independently with ClustalW [20] and the gap-including sites at both ends were pruned using MEGA 10.2 software [21]. ML analyses were performed using MEGA 10.2 software. The best substitution model for ML analysis was decided based on the Akaike information criterion in the complete edition of the sequence using model search; the Tamura-Nei model + G was chosen for the substitution model. All gap-including sites were eliminated from the dataset, after which bootstrap (BS) analysis was performed in MEGA 10.2 software.

Bayesian probability (BP) analysis was performed using MrBayes (version 3.1.1) [22]. The best-fit models were separately searched for each DNA region using MrModeltest v.2.3 [23], and performed using PAUP* v.4.0b10 [24]. SYM + G, HKY + I, and HKY + G were selected for ITS, *GAPDH*, and *CHS1*, respectively, while SYM + I was selected for *ACT* and *TUB2*. Analysis of the two Markov and Monte Carlo chains of the four runs was run for 1,000,000 generations. Data were rated every 100 generations, and the first 6500 generations were burned-in, because the average standard deviation of the split frequency rate in these generations was less than 0.01.

### 2.5. Pathogenicity Test

Pathogenicity tests were conducted using a conidial suspension adjusted to 1 × 10^5^ conidia/mL. Twenty microliters of the conidial suspension were dropped onto three areas per pod, some of which had wounds, and some did not, on healthy young and mature cacao pods, and on young and old leaves. Sterile water was used as control. The samples were covered with plastic bags for five days to maintain high humidity. After 9–14 d, the inoculated samples were observed, and re-isolation was conducted from symptomatic pods and leaves.

### 2.6. Mycelial Growth Test

To determine the optimal temperature for the growth of the isolate, a mycelial growth test was conducted twice with 5 replicates each. The mycelial plugs (φ6 mm) were obtained from the colonies of our isolate, which was grown for 7 d at 25 °C, and placed at the center of PDA, SNA, and OA plates (φ9 cm). The plates were incubated at 4, 10, 15, 20, 25, 30, 35, and 40 °C for 4 d. Five replications were conducted for each temperature. Mycelial growth was measured daily, and the average colony diameter at each temperature was calculated.

## 3. Results

### 3.1. Molecular Phylogenetic Analyses

To clarify the molecular phylogenetic position of our isolate, a BLAST search was performed in the NCBI database, and phylogenetic analyses were conducted. Partial nucleotide sequences of ITS, *GAPDH*, *CHS1*, *ACT*, and *TUB2* were matched with *C. gigasporum* strains JS-0328 (query cover: 99%; 550 bp; identity: 100%; accession no. OM397116), CBS 132881 (query cover: 100%; 295 bp; identity: 99%; accession no. KF687829), JS-0367 (query cover: 100%; 727 bp; identity: 95%; accession no. CP077955), JS-0367 (query cover: 100%; identity 94%; accession no. CP077947), and CX34 (query cover 100%, 678 bp; identity 99%; accession no. ON420414). These species, which were identified using the nucleotide data of our isolate, belong to CGISC.

**Figure 2:**
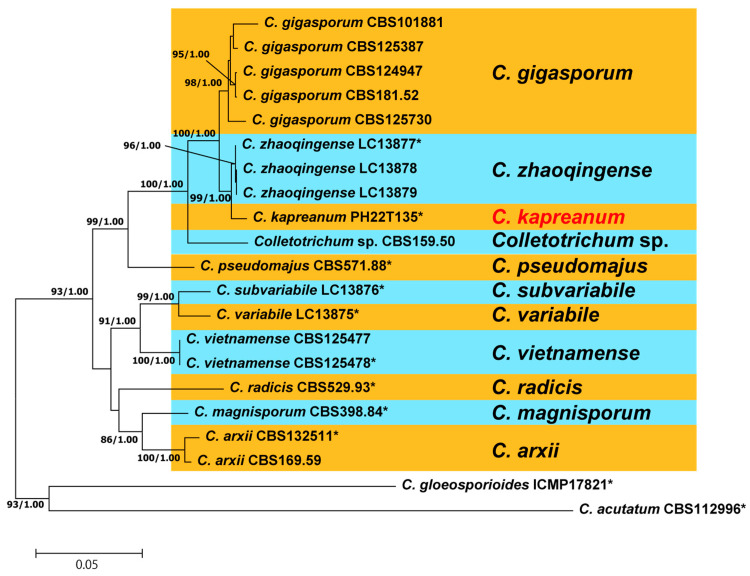
Maximum likelihood (ML) tree of the *Colletotrichum gigasporum* species complex based on combined data sets of ITS, *GAPDH*, *CHS1*, *ACT*, and *TUB2* sequences (1725 bp including gaps). ML bootstrap values and BP analysis are shown at the nodes (BS/BP). BS > 80% and BP > 0.95 are shown. Asterisks indicate ex-type strains. *C. kapreanum*, written in red, is a new species proposed in the present study.

Identification of our isolate at the species level was conducted using the data of species in CGISC (Figure 2). The final dataset contained 1725 bp, including gaps, comprising 545, 286, 280, 228, and 391 positions from ITS, *GAPDH*, *CHS1*, *ACT*, and *TUB2*, respectively. As the ML and BP trees exhibited similar topologies, the ML tree shown in Figure 2 had the highest log likelihood score of −6928.70. Our isolate was independent from the other 11 clades, including all 9 ex-type strains belonging to CGISC (Figure 2), and most closely related to *C. zahaoqingense*, as evidenced by high BS values and BP (BS/BP: 99/1.0).

### 3.2. Pathogenicity Test

**Figure 3:**
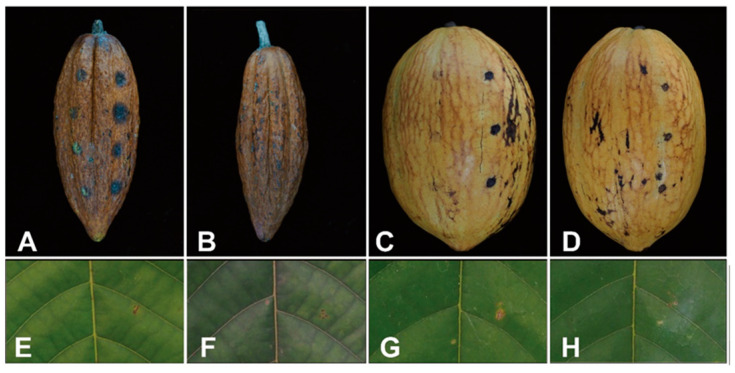
Cacao pods and leaves inoculated with the PH22T135 strain. Black spot lesions in (**A**) wounded (right) and unwounded (left) cherelle and (**B**) the control nine days after inoculation. (**C**) Black spots appeared in the mature wounded pod (right) whereas no spots were found in the unwounded pod (left) 14 days after inoculation or (**D**) control. No lesions were found in (**E**) wounded (right) and unwounded (left) or (**F**) control young leave, nor in (**G**) wounded or (**H**) control old leave at 14 days after inoculation.

Our isolate produced symptoms in cherelles, resulting in black lesions in all inoculated areas four days after inoculation (Figure 3A). In mature pods, black spots appeared only in wounded areas (Figure 3C). These were slightly darker than those in control (Figure B), and no expansion of lesions was observed. No symptoms developed in the pods or leaves treated with sterile water as controls (Figure 3B,D,F,H). The inoculum was re-isolated from all symptomatic tissues. Regarding pathogenicity on wounded young and mature leaves, the isolate did not show pathogenicity as seen in cherelles (Figure 3E,G). However, over time, the inoculated parts were slightly bigger as lesions than the control one, and the inoculum was re-isolated from the lesion.

### 3.3. Taxonomy

***Colletotrichum kapreanum*** Takata & Kyoko Watan., sp. nov. (Figure 1).

MycoBank no.: MB 852300.

Etymology: The species name was derived from the name ‘kapre’, a giant living on a tree based on Philippine mythology.

Type: Machida, Tokyo, Japan, Tamagawa University, isolated from a wilted cacao cherelle. Holotype PH22T135S, ex-type strain PH22T135.

**Figure 4:**
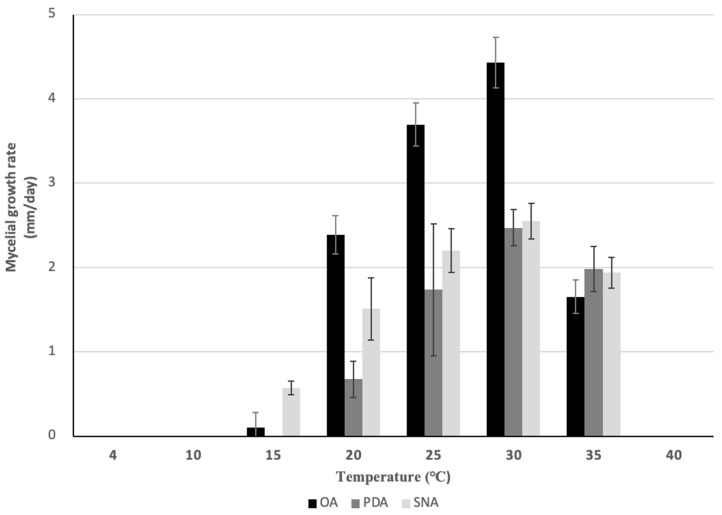
Mycelial growth rate of *C. kspresii* PH22T135 on OA, PDA, and SNA at different temperatures in the dark for 7 days. The vertical lines on each bar indicate standard deviations.

Sexual morph: Sexual morphology was observed in colonies grown on OA plates, exclusively on black hyphae (Figure 1B). Ascomata were solitary, scattered, and few. Asci were unitunicate, eight-spored, broadly cylindrical to clavate, and truncate at apex (Figure 1G,H). Ascospores were hyaline, fusoid to cylindrical, mostly straight to slightly curved or simultaneously irrigate curved, rounded at both ends, aseptate or 1-septum (rarely 3 septa), septum median, and measured (44.5–)47.5–68.6(–69.5) × 5.2–7.0(–7.4) μm (ave. ± sd. 60.5 ± 6.5 × 6.0 ± 0.5) (Figure 1I–L). Asexual morph: Colony grew on OA plates at 15–35 °C with an optimum temperature of 30 °C (ave. ± sd. 4.4 ± 0.3 mm/day) in the dark (Figure 4), davis gray to sonic silver. Hyphae were hyaline, smooth-walled, septate, and branched. Setae were medium brown, verruculose, 6–8-septae, and 70–160 μm long. Conidiomata was pale orange (Figure 1C). Conidia were aseptate straight, cylindrical, guttulate, rounded at both ends, and measured 22.9–28.4 × 7.1–8.7 (ave. ± sd. 26.2 ± 1.7 × 8.1 ± 0.4) μm (L/W = 3.2) (Figure 1M). On PDA plates, hyphae grew at 20–35 °C, with an optimum temperature of 30 °C (ave. ± sd. 2.5 ± 0.2 mm/day) in the dark. Conidia were not observed. On SNA, hyphae grew at 15–35 °C, with an optimum temperature of 30 °C (ave. ± sd. 4.4 ± 0.3 mm/day) in the dark. Conidia were not observed. Appressoria were pale to medium brown, irregular or ovoid-shaped, and measured 4.9–9.6(–10.8) × (2.9–)3.1–5.1(–7.6) μm (ave. ± sd. 10.8 ± 1.5 × 4.1 ± 0.9) (L/W = 1.8) (Figure 1N–Q). On pine needles, conidiomata were pale orange and there were few (Figure 1D). The acervalus was lined with conidiogenous cells (Figure 1E). Setae were 6–8 septate, medium brown, verruculose, 6–8-setae, 80–140 μm long. Conidia were aseptate, straight, cylindrical, guttulate, rounded at both ends, and measured (18.2–)21.2–25.5 × 6.7–7.1 (ave. ± sd 22.2 ± 1.6 × 6.9 ± 0.4) μm (Figure 1F).

Note: *C. kapreanum* resembles *C. zahaoqingense*, a sister group of *C. kapreanum*, in the shape of conidia and appressoria, but not in conidial size, as those of *C. kapreanum* are larger than those of *C. zahaoqingense* (Table 2). Two species produce ascospores with septa in the CGISC, including *C. gigasporum* [18,25] and *C. taiwanense* [26]; however, the number of septa in *C. taiwanense* is larger than that of *C. kapreanum* (Table 3). The size of the conidia of *C. kapreanum*, however, is similar to that of *C. gigasporum* (Table 2). Meanwhile, the size of appressoria of *C. gigasporum* is larger than those of *C. kapreanum* (Table 4). Recorded sizes of ascospores of *C. gigasporum* and *C. taiwanense* were measured with PDA plates and rice straw, but PH22T135 asci and ascospores were not observed on PDA plates.

## 4. Discussion

### 4.1. Identification of Pathogenic Isolate

The PH22T135 strain extracted from cherelle wilt was identified as a new species pathogenic to cacao, belonging to the CGISC based on morphological and phylogenetic analyses; it was named *Colletotrichum kapreanum*. Although there are some reports of other pathogens belonging to the genus *Colletorichum* that affect cacao, *C. kapreanum* is independent of not only these species, including *C. gloeosporioides*, *C. siamense*, *C. theobromicola*, *C. tropicale*, and *C. aeschynomenes* [4,6,7,8,9], but also other reported species in the molecular phylogenetic tree, per morphological analysis. CGISC comprises 17 species, typically with longer conidia, characterized by a straight shape with a round end, than those of other *Colletotrichum* species, belonging to other species complexes. These species can also be distinguished from other species in other species complexes using phylogenetic analyses [19,20]. The anamorph of *C. kapreanum* is consistent with the morphology of species belonging to CGISC. Focusing on morphologies of conidia and appressoria, *C. kapreanum* is distinct from *C. zhaoqingense* in the size of conidia and the shape and size of appressoria. Although *C. gigasporum* produces similarly sized conidia to that of *C. kapreanum*, their appressoria are significantly different in size. Additionally, we morphologically compared *C. kapreanum* with *C. taiwanense*, the latter of which has no molecular data, because the anamorphs of *C. taiwanense* are alike those of the species in CGISC, and we found no significant difference in the size of conidia between *C. kapreanum* with *C. taiwanense*. However, these two species also are distinguished from each other in the size and shape of appressoria. Regarding morphologies of ascospores, *C. kapreanum* produces smaller ascospores than those of *C. gigasporum*, while those produced by *C. taiwanense* are similar in size to those of *C. kapreanum*. However, *C. kapreanum* differs from *C. taiwanense* in terms of the number of septa of ascospores. *C. kapreanum* produces ascospores with a septum in the middle (usually one septum, rarely three septa), while *C. taiwanense* produces 0–3 ascospores and, rarely, eight septa [26,27]. Therefore, we propose *C. kapreanum* as the new species belonging to CGISC. To the best of our knowledge, this is the third report of this species having ascospores with a septum, as belonging to the genus *Colletotrichum*.

### 4.2. Pathogenicity

The isolate was relatively pathogenic toward cherelles and produced symptoms in both wounded and unwounded parts of the organ. However, the isolate did not show pathogenicity to mature pods and leaves. *C. tropicale* is reported to cause anthracnose of cacao pods at different stages, including young, immature, and mature pods [5]. Paguntalan et al. [27] reported *Colletotrichum* sp. isolated from an anthracnose-infected pod through a postharvest survey in Mindanao, Philippines. This strain belongs to CGLSC, according to the results obtained from morphological data and the pictures in their article. There are no records of *Colletotrichum* spp., except for CGLSC, related to anthracnose in cacao. In addition to our results, cacao pods are affected by different pathogens depending on their developmental stage which is a point to be noted when managing the relevant diseases.

In the life cycle of the plant pathogens, latent infection must be considered because this information is crucial in disease management strategies. Some studies provide information on the importance of latent infection of *Colletotrichum* spp. to control the disease. For example, Peres et al. [12] investigated the life cycle of the pathogenic *C. acutatum* in citrus, strawberries, blueberries, and almonds, which was detected as the causal agent of latent infection stages in the tissues of leaves, twigs, and/or flowers. Eventually, these fungi produced fruit rot from acervuli that appeared on damaged tissues, infected twigs, flowers, and/or buds. Our isolate *C. kapreanum* produced symptoms on mature pods, and colonies could be reisolated, which indicates that, although our isolate can be regarded as a host organ-specific pathogen to cherelles, it may be able to infect leaves and mature pods quiescently. However, knowledge about the biotrophic and epiphytic phases of this pathogen is needed. Furthermore, the investigation of this species on several organs with/without symptoms in cacao trees is essential for clarifying the life cycle of the pathogen, and further investigation of pathogens causing cherelle wilt in cacao could help clarify whether *C. kapreanum* is the dominant pathogen of cacao cherelles.

## 5. Conclusions

A new fungal pathogen, *Colletotrichum kapreanum* sp. nov, was identified in cacao fields in Pangasinan, Philippines. This pathogen only affects cacao cherelles, not pods, and can lead to a reduction in cacao production. Removing diseased cherelles is crucial in mitigating its impact on cacao cultivation.

## Figures and Tables

**Table 1:** Details of strains included in the phylogenetic analyses.

Species	Strain	Species Complex	Host	GenBank Accession Number				
				ITS	*GAPDH*	*CHS1*	*ACT*	*TUB2*
*C. arxii*	CBS169.59	Gigasporum	*Oncidium excavatum*	KF687717	KF687824	KF687781	KF687784	KF687868
*C. arxii*	Paphi 2-1 *	Gigasporum	*Paphiopedilum* sp.	KF687716	KF687843	KF687780	KF687802	KF687881
*C. gigasporum*	CBS101881	Gigasporum	*Solanum betaceum*	KF687736	KF687841	KF687777	KF687797	KF687886
*C. gigasporum*	CBS124947	Gigasporum	*Theobroma cacao*	KF687731	KF687828	KF687763	KF687786	KF687871
*C. gigasporum*	CBS125385	Gigasporum	*Theobroma cacao*	KF687733	KF687834	KF687765	KF687788	KF687873
*C. gigasporum*	CBS125730	Gigasporum	*Theobroma cacao*	KF687735	KF687840	KF687770	KF687793	KF687878
*C. gigasporum*	CBS181.52	Gigasporum	*Theobroma cacao*	KF687734	KF687838	KF687775	KF687799	KF687885
*C. magnisporum*	CBS398.84 *	Gigasporum	Unknown	KF687718	KF687842	KF687782	KF687803	KF687882
*C. pseudomajus*	CBS571.88 *	Gigasporum	*Camellia sinensis*	KF687722	KF687826	KF687779	KF687801	KF687883
*C. radicis*	CBS529.93 *	Gigasporum	Unknown	KF687719	KF687825	KF687762	KF687785	KF687869
*C. subvariabile*	NN040649 *	Gigasporum	Unknown plant	MZ595883	MZ664054	MZ799343	MZ664181	MZ674001
*C. variabile*	NN040656 *	Gigasporum	Unknown plant	MZ595884	MZ664055	MZ799344	MZ664182	MZ674002
*C. vietnamense*	CBS125477	Gigasporum	*Coffea* sp.	KF687720	KF687831	KF687768	KF687791	KF687876
*C. vietnamense*	LD16(L2) *	Gigasporum	*Coffea* sp.	KF687721	KF687832	KF687769	KF687792	KF687877
*C. zhaoqingense*	NN058985 *	Gigasporum	Dead petiole of palm	MZ595905	MZ664065	MZ799304	MZ664203	MZ674023
*C. zhaoqingense*	NN071035	Gigasporum	Dead petiole of palm	MZ595906	MZ664066	MZ799305	MZ664204	MZ674024
*C. zhaoqingense*	NN071036	Gigasporum	Dead petiole of palm	MZ595907	MZ664067	MZ799306	MZ664205	MZ674025
***C. kapreanum***	PH22T135 *	**Gigasporum**	***Theobroma cacao*, cherelle**	**LC750347**	**LC750348**	**LC750349**	**LC750350**	**LC750351**
*Colletotrichum* sp.	CBS159.50	Gigasporum	*Derris* sp.	KF687724	KF687823	KF687778	KF687800	KF687867
*C. acutatum*	CBS112996 *	Acutatum	*Carica papaya*	JQ005776	JQ948677	JQ005797	JQ005839	JQ005860
*C. gloeosporioides*	ICMP17821 *	Gloeosporioides	*Citrus sinensis*	JX010152	JX010056	JX009818	JX009531	JX010445

The new sequences obtained in this study are highlighted in bold. * Ex-type cultures.

**Table 2:** Conidial characteristics of *Colletotrichum kapreanum* and related species.

Species	Strain	Conidial Size (µm)			Medium
		Size (µm)	Ave. ± SD (µm)	L/W Ratio	
*C. kapreanum*	TAP22T135	22.9–28.7 × 7.1–8.7	26.2 ± 1.7 × 8.1 ± 0.4	3.2	OA
		(18.2–)21.2–25.5 × 6.7–7.1	22.2 ±1.6 × 6.9 ± 0.4	3.0	Pine needle
*C. gigasporum* ^a^	CBS133266	(22–)25–29(–32) × (6–)7–9	NR	NR	PDA
*C. zhaoqingense* ^b^	NN058985	20–24 × 5.5–7	21.3 ± 0.9 × 6.3 ± 0.6	3.1	Pine needle
*C. taiwanense* ^c^	IMI353024	22–35(–45) × 5–8	NR	NR	PDA

^a^ Rakotoniriana et al. [25], ^b^ Liu et al. [18], ^c^ Sivanesan and Hsieh [26]. NR denotes data not reported.

**Table 3:** Ascospore characteristics of *Colletotrichum kapreanum* and related species.

Species	Strain	Ascospores		Medium
		Size (µm)	No. of Septa	
*C. kapreanum*	TAP22T135	(44.5–)47.5–68.6(–69.5) × 5.2–7(–7.4)	0–3 (mainly 1)	OA
*C. gigasporum* ^a^	CBS133266	(56–)60–84(–92) × 5–7	0–1	PDA
*C. taiwanense* ^b^	IMI353024	50–58 × 5–8	4–5	Rice straw

^a^ Rakotoniriana et al. [25], ^b^ Sivanesan and Hsieh [26].

**Table 4:** Appressorial characteristics of *Colletotrichum kapreanum* and related species.

Species	Strain	Appressoria		
		Size (µm)	Color	Shape
*C. kapreanum*	TAP22T135	4.9–9.6(–10.8) × (2.9–)3.1–5.1(–7.6)	pale to medium brown	irregular, ovoid
*C. gigasporum* ^a^	CBS133266	14–16 × 10–12	pale to medium brown	clavate, irregular
*C. zhaoqingense* ^b^	NN058985	5.5–18 × 3.5–6.5	pale brown	globose, subglobose, ovoid, irregular
*C. taiwanense* ^c^	IMI353024	7.0–12.5 × 7.5–15.5	brown	globose, clavate, irregular

^a^ Rakotoniriana et al. [25], ^b^ Liu et al. [18], ^c^ Sivanesan and Hsieh [26].

## Data Availability

DNA data obtained in this study were submitted to the DNA data bank of Japan, and their accession numbers are found in Table 1.
